# Epidemiological analysis of Lambert-Eaton myasthenic syndrome in Türkiye: insights from a nationwide electronic health database

**DOI:** 10.3389/fneur.2025.1667540

**Published:** 2025-08-25

**Authors:** Berin Inan, Bilgin Ozturk, Naim Ata, Suayip Birinci, Erdal Eroglu, Omer Karadas, Ersin Tan, Zeki Odabasi

**Affiliations:** ^1^Department of Neurology, Gulhane Medical Faculty, University of Health Sciences, Ankara, Türkiye; ^2^General Directorate of the Health Information Systems, Republic of Türkiye Ministry of Health, Ankara, Türkiye; ^3^Republic of Türkiye Ministry of Health, Ankara, Türkiye; ^4^Department of Neurology, Faculty of Medicine, Hacettepe University, Ankara, Türkiye

**Keywords:** Lambert-Eaton myasthenic syndrome, epidemiology, incidence, prevalence, autoimmune, paraneoplastic, small cell lung cancer

## Abstract

**Introduction:**

Lambert-Eaton myasthenic syndrome (LEMS) is a rare autoimmune disorder of the neuromuscular junction, with limited large-scale epidemiological data. In this study, we aimed to determine the epidemiological profile of LEMS in Türkiye, and to assess associated malignancies, mortality, and prescription rates of pyridostigmine and amifampridine.

**Methods:**

We identified LEMS cases through a retrospective review of clinical records for individuals with a G73.1 code entry in the national healthcare database between 2015 and 2024. Confirmed cases were classified as autoimmune (A-LEMS) or paraneoplastic (P-LEMS). Demographic, clinical, and prescription data were analyzed, and incidence and prevalence rates were calculated using official census data.

**Results:**

A total of 159 LEMS cases were confirmed. The median age at diagnosis was 60 years, and 55.3% of the patients were female. P-LEMS accounted for 59.7% of cases, with small cell lung cancer (SCLC) present in 55.8% of these. Annual incidence of LEMS ranged from 0.09 to 0.30 per million, and the overall 2024 prevalence was 1.11 per million. A-LEMS had a higher prevalence than P-LEMS in 2024, likely due to its lower mortality (23.4% vs. 58.9%). P-LEMS was more common in older males and predominantly associated with SCLC. Pyridostigmine was prescribed to 65.4% of patients, and amifampridine to 24.5%, with both treatments more frequently used in A-LEMS.

**Discussion:**

This is the first nationwide epidemiological study of LEMS in Türkiye, revealing lower incidence and prevalence rates than in other countries. This study provides valuable large-scale epidemiological data, enriching the global understanding of this rare disorder.

## 1 Introduction

Lambert-Eaton myasthenic syndrome (LEMS) is a rare autoimmune disorder of the neuromuscular junction, characterized by antibodies targeting presynaptic voltage-gated calcium channels, which leads to reduced acetylcholine release from nerve terminals and subsequent impaired neuromuscular transmission (1). The clinical presentation of LEMS typically includes proximal muscle weakness, diminished or absent deep tendon reflexes, and various autonomic symptoms such as dry mouth, and constipation (2, 3).

LEMS can be classified into two distinct forms: paraneoplastic (P-LEMS) and autoimmune (A-LEMS) subtypes (4). Nearly half of all LEMS cases are associated with an underlying malignancy, predominantly small cell lung cancer (SCLC) (5). Studies suggest that LEMS is observed in approximately 3% of patients with SCLC (6), and conversely, 40–70% of individuals diagnosed with LEMS are subsequently found to have SCLC (7–9).

Although the global incidence and prevalence rates of LEMS remain uncertain, population-based studies have provided some insight. A study conducted in the Netherlands reported an incidence of 0.5 and a prevalence of 2.3 per million (10). Similarly, a United States Veterans Affairs population-based study reported a prevalence rate of 2.6 per million (11). Although LEMS can affect individuals across all age groups, it predominantly occurs in middle-aged adults. Notably, A-LEMS tends to present at a younger age than P-LEMS (7).

The primary objective of this study was to determine the epidemiological profile of LEMS in Türkiye. Secondarily, we aimed to assess the associated malignancies, mortality, and prescription rates of pyridostigmine and amifampridine among patients with LEMS.

## 2 Materials and methods

In Türkiye, national healthcare data are mainly managed by the Republic of Türkiye Ministry of Health (RTMH) through the Health Record Reporting System (HRRS) and e-Nabiz platform. HRRS is a platform used to systematically document and report healthcare data for health services management, whereas e-Nabiz functions as an integrated digital health interface, allowing both patients and healthcare providers to access medical records gathered from various healthcare facilities.

Working in collaboration with the Health Policy Development Working Group of the RTMH, we initially identified individuals with G73.1 diagnostic code entries, corresponding to LEMS in the International Classification of Diseases, 10th revision (ICD-10), in the RTMH electronic database between January 1, 2015, and December 31, 2024. Given that diagnostic codes in this system are retained indefinitely, regardless of whether they reflect preliminary assessments, confirmed diagnoses, or are used for medication purposes, we retrospectively reviewed clinical documentation of each case to verify the diagnosis. Only cases supported by sufficient evidence, whether clinical, electrophysiological, or laboratory-based, were considered “confirmed” cases and included in the study cohort. The confirmed cases were further subclassified as either A-LEMS or P-LEMS. P-LEMS was defined by the presence of a cytologically or histopathologically confirmed malignancy documented in the national health registry within a clinically relevant timeframe. A-LEMS referred to cases with no associated malignancy. Throughout the study, all data were anonymized, and data handling adhered strictly to national data protection regulations.

Demographic and clinical data, including sex, date of birth, age at diagnosis, province of residence, date of death (if applicable), and associated malignancies based on cytology or pathology reports were collected. The date of diagnosis was defined as the date of the first G73.1 entry in the RTMH database. Prescription data for amifampridine and pyridostigmine were obtained using their Anatomical Therapeutic Chemical codes, N07XX05 and N07AA02, respectively.

Incidence rates were calculated annually between 2015 and 2024 for the overall population, while the prevalence rate was calculated for 2024 both at the national level and across Türkiye’s seven geographical regions. National population data, including sex- and province-specific figures, were obtained from the Turkish Statistical Institute website (12) for the corresponding years.

This study was conducted in accordance with the Declaration of Helsinki. Both the RTMH and the institutional ethics committee approved the study (2024-438, 10/09/2024).

The normality of the data was assessed using the Kolmogorov–Smirnov test. Descriptive statistics were expressed as mean ± standard deviation for normally distributed continuous variables, and as median (minimum–maximum) for non-normally distributed data. Categorical variables were presented as frequencies and percentages. We compared independent groups using the Mann–Whitney U test. The Chi-square test was used to evaluate the associations between categorical variables. IBM SPSS Statistics for Windows, version 23.0 (IBM Corp., Armonk, NY, USA) was used for statistical analyses. Data visualization was performed with GraphPad Prism, version 10.2.3 for Mac (GraphPad Software, Boston, MA, United States).^1^ A *p*-value < 0.05 was considered indicative of statistical significance.

## 3 Results

**Figure 1:**
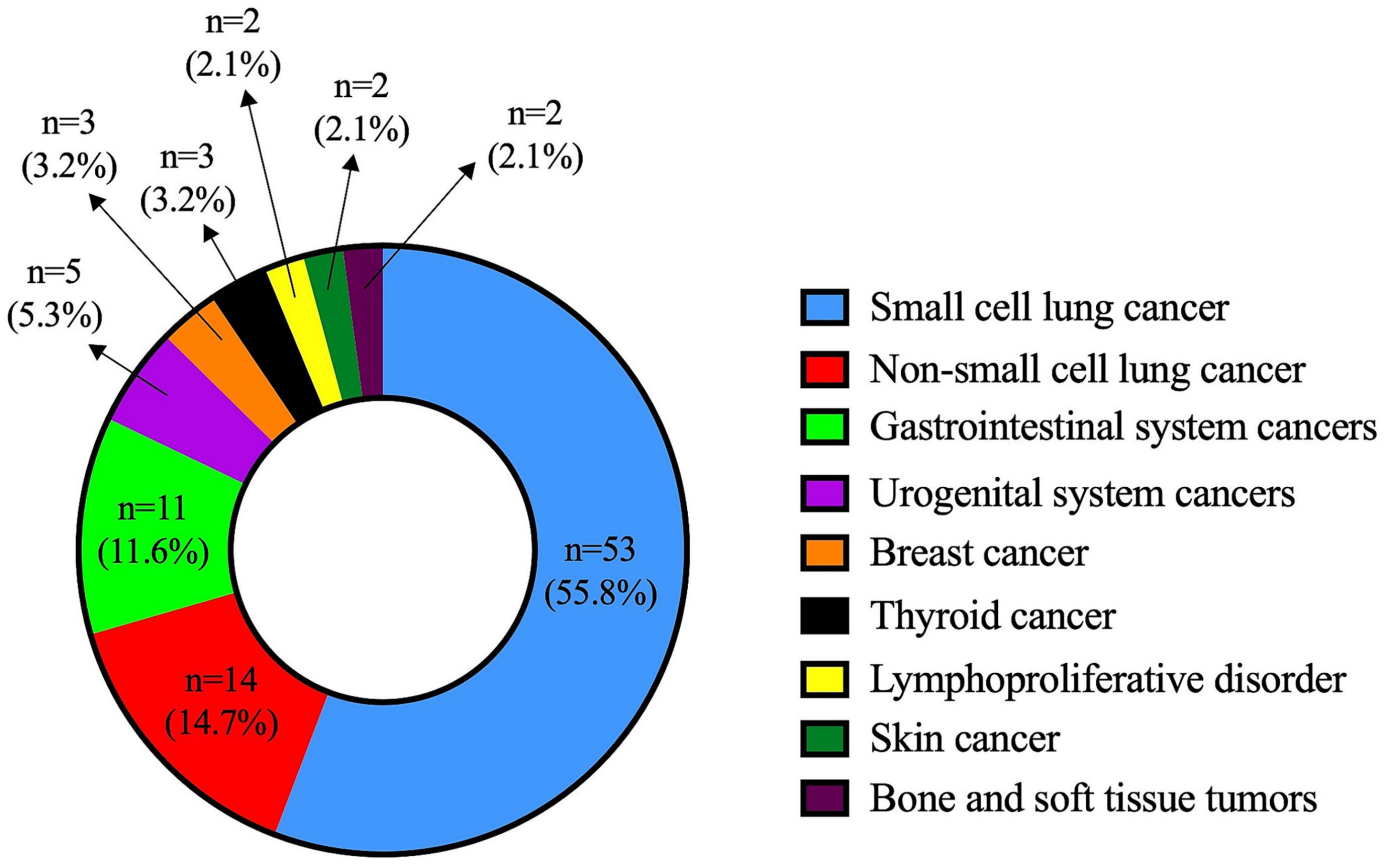
Distribution of malignancy types in patients with paraneoplastic Lambert-Eaton myasthenic syndrome.

Türkiye is a large country with a total population of 85,664,944 according to the 2024 national census data. Initial screening of the national healthcare database revealed 7,770 individuals with at least one entry of the G73.1 diagnostic code. Following a review of clinical documentation, we identified 159 confirmed LEMS cases. Eighty-eight (55.3%) of them were female, and the female-to-male ratio was 0.8. The median age at diagnosis was 60.0 (16.0–88.0) years (mean: 58.1 ± 14.9). Most of the patients (*n* = 95, 59.7%) had P-LEMS, with 55.8% (*n* = 53) of them being associated with SCLC. The distribution of all associated malignancies is presented in Figure 1.

Pyridostigmine was prescribed to 104 patients (65.4%), and amifampridine to 39 patients (24.5%) with LEMS. Seventy-one (44.7%) patients had died by the end of 2024. A comparison between A-LEMS and P-LEMS subgroups is presented in Table 1.

**Table 1:** Comparison of patients with A-LEMS and P-LEMS.

Characteristics	A-LEMS	P-LEMS	*p*-value
Females (*n*, %)	38 (59.4%)	33 (34.7%)	**0.02**
Age at diagnosis, median (min-max)	54.5 (16.0–82.0)	62.0 (18.0–88.0)	**<0.001**
Mortality (*n*, %)	15 (23.4%)	56 (58.9%)	**<0.001**
Amifampridine prescription (*n*, %)	25 (39.1%)	14 (14.7%)	**<0.001**
Pyridostigmine prescription (*n*, %)	52 (81.3%)	52 (54.7%)	**0.001**

A-LEMS, autoimmune Lambert-Eaton myasthenic syndrome; P-LEMS, paraneoplastic Lambert-Eaton myasthenic syndrome.Bold values indicate statistical significance (*p* < 0.05).

**Figure 2:**
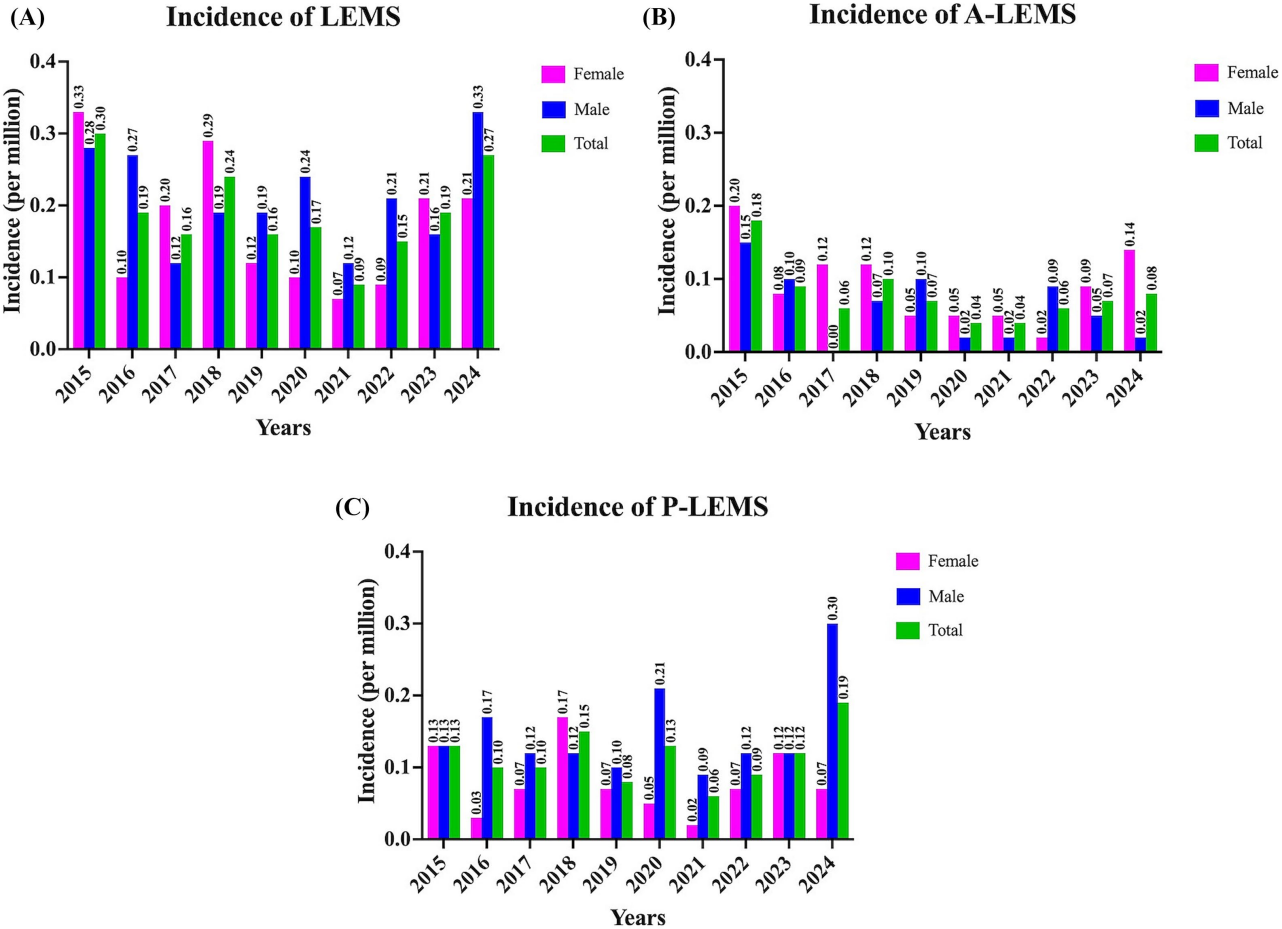
Incidence rates of Lambert-Eaton myasthenic syndrome for 2015–2024 years. **(A)** Overall incidence rates of Lambert-Eaton myasthenic syndrome (LEMS); **(B)** Annual incidence of autoimmune LEMS (A-LEMS); and **(C)** Annual incidence of paraneoplastic LEMS (P-LEMS) per million population stratified by sex. The data reflect variations in incidence trends across the 10-year period.

Between 2015 and 2024, the annual incidence of LEMS ranged from 0.09 to 0.30 per million. The incidence of A-LEMS varied between 0.04 and 0.18 per million, while that of P-LEMS ranged from 0.06 to 0.19 per million. Incidence rates of P-LEMS were higher than those of A-LEMS in all years except 2015. A detailed overview of incidence trends is provided in Figure 2.

**Figure 3:**
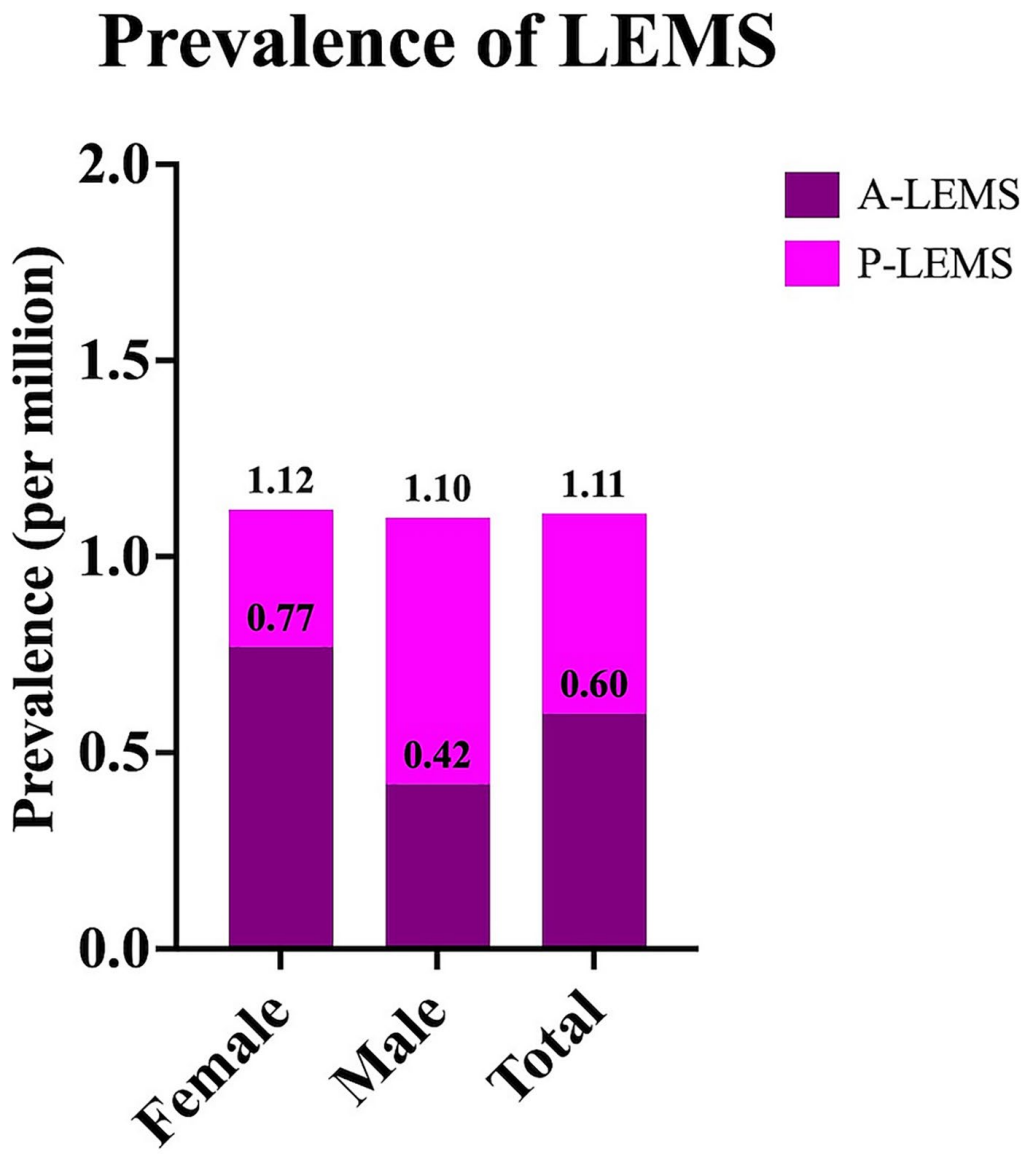
Prevalence rates of Lambert-Eaton myasthenic syndrome and its subtypes in Türkiye in 2024. Prevalence rates of Lambert-Eaton myasthenic syndrome (LEMS), autoimmune LEMS (A-LEMS), and paraneoplastic LEMS (P-LEMS) are presented as sex-specific and total values.

The overall prevalence of LEMS was calculated as 1.11 per million, with A-LEMS and P-LEMS accounting for 0.60 and 0.51 per million, respectively. Sex-specific prevalence rates are presented in Figure 3.

**Figure 4:**
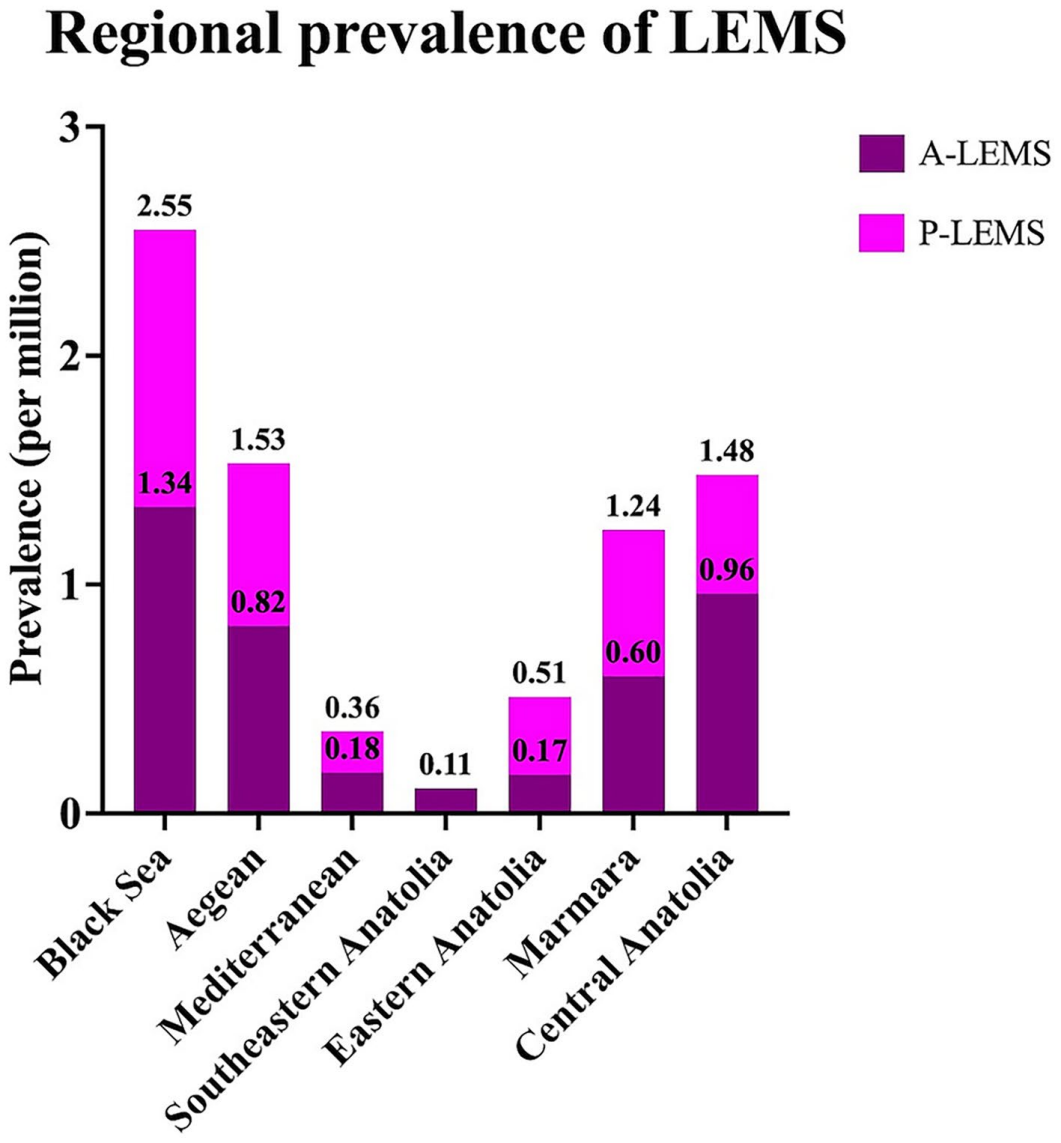
Regional prevalence rates of Lambert-Eaton myasthenic syndrome and its subtypes in 2024. A-LEMS, autoimmune Lambert-Eaton myasthenic syndrome; P-LEMS, paraneoplastic Lambert-Eaton myasthenic syndrome.

The highest prevalence rate of LEMS was observed in the Black Sea region, while Southeastern Anatolia exhibited the lowest prevalence rate (Figure 4). The cities with the highest number of LEMS cases were Istanbul, Ankara, and Izmir, respectively.

## 4 Discussion

This nationwide study represents the first comprehensive epidemiological analysis of LEMS in Türkiye based on the national electronic health database, and one of the few large, population-based studies in the literature (10, 11, 13–15).

The annual incidence of LEMS in Türkiye ranged from 0.09 to 0.30 per million between 2015 and 2024, with a rate of 0.27 per million in 2024. These rates were slightly lower than those reported in the Netherlands (0.40 and 0.48 per million) (10, 13) and the United States Veterans Affairs (0.6 per million) (11) studies. Similarly, the overall prevalence of LEMS in Türkiye (1.11 per million) was notably lower compared to previous estimates from the Netherlands (2.32 and 2.50 per million) (10, 13), the United States (2.6 per million) (11), Ireland (2.9 per million) (14) and Japan (2.7 per million) (15). This discrepancy may be attributed to multiple factors, including possible underdiagnosis due to limited disease awareness, differences in access to healthcare services, variability in clinical suspicion, and disparities in registry completeness and reporting practices. Future prospective, multicenter, or international studies are needed to clarify these epidemiological differences.

In our cohort, 55.3% of the patients were female, contrasting with the slight male predominance reported in other studies (8, 10, 15, 16). An exception was the United States Veterans Affairs study (11), which reported 98% male patients, reflecting the predominantly male veteran population. The median age at diagnosis in our cohort was 60 years, which is slightly higher than the median ages reported in studies from the Netherlands (10) and China (8).

P-LEMS accounted for approximately 60% of cases in our cohort. This finding is consistent with reports from the United States Veterans Affairs (11), China (8) and the Netherlands (16), but lower than the proportion reported in another Dutch study (13) and higher than those observed in Ireland (14), Japan (15), and another national cohort study conducted in the Netherlands (10). We observed a clear demographic divergence between P-LEMS and A-LEMS in our cohort. In line with previous studies, P-LEMS was more frequent in males and older individuals, whereas A-LEMS occurred more commonly in younger female patients (2, 10, 15, 16). The higher prevalence of P-LEMS among older males likely reflects its strong association with SCLC, which is more common in this demographic (17, 18). In contrast, the predominance of A-LEMS in younger females aligns with gender trends commonly observed in autoimmune diseases (19). The presence of SCLC significantly influences both the prognosis and the treatment approach in patients with LEMS. For this reason, scoring systems have been developed to estimate the risk of SCLC in individuals diagnosed with LEMS (2, 20, 21). Clinical predictors associated with an increased likelihood of underlying SCLC include: age at onset ≥50 years, smoking at the time of diagnosis, weight loss of ≥5%, bulbar symptoms, erectile dysfunction, and a Karnofsky performance status score of less than 70 (2). Meanwhile, patients with A-LEMS are at elevated risk for other organ-specific autoimmune diseases (22, 23). Recognizing these demographic and clinical patterns is essential for identifying high-risk patients, minimizing diagnostic delays for malignancies, and optimizing both tumor screening protocols and individualized treatment strategies.

As expected, SCLC was the most frequently associated malignancy in P-LEMS, found in 55.8% of cases. This was consistent with previous studies reporting SCLC as the leading cancer type in this subgroup (8, 10, 11, 15).

Although the annual incidence of P-LEMS consistently exceeded that of A-LEMS in all years except 2015, the prevalence of A-LEMS surpassed that of P-LEMS in 2024. This may reflect the substantially higher mortality associated with P-LEMS (58.9%) compared to A-LEMS (23.4%), a pattern also reported in cohorts from the Netherlands and Japan (10, 15). The aggressive nature of SCLC and other associated malignancies likely contributes to shortened survival among P-LEMS patients (10, 11, 16).

The regional prevalence analysis in our cohort revealed considerable geographic variation, with the highest prevalence observed in the Black Sea, Aegean, and Central Anatolia regions. These disparities may relate to regional differences in smoking prevalence, environmental exposures, or diagnostic practices, though further studies are warranted to clarify these factors.

Amifampridine and pyridostigmine are widely used for the symptomatic treatment of LEMS, either as monotherapy or in combination (24). Amifampridine exerts its effect by blocking presynaptic potassium channels, thereby prolonging depolarization and enhancing calcium influx. This results in increased acetylcholine (Ach) release at the neuromuscular junction (25). Amifampridine is the first drug of choice for symptomatic management of LEMS, as it is more effective than pyridostigmine in improving muscle strength and electrophysiological parameters (24, 26–28). However, when amifampridine is not readily accessible or is poorly tolerated, pyridostigmine is used (25). Pyridostigmine enhances Ach availability at the neuromuscular junction by inhibiting its enzymatic breakdown (24). In our cohort, pyridostigmine was prescribed to 65.4% of patients, and amifampridine to 24.5%. The pyridostigmine prescription rate was comparable to those reported in studies from the Netherlands (16) and Japan (15), but lower than that observed in the United States cohort (11). The amifampridine prescription rate in our cohort was considerably lower than those reported in Japan, the US Veterans Affairs population, and the Netherlands (11, 15, 16), where amifampridine usage reached as high as 95% (16). Notably, A-LEMS patients in our cohort were significantly more likely to receive both medications than those with P-LEMS, consistent with findings from the Japanese cohort (15). In Türkiye, amifampridine is imported on a case-by-case basis following formal application and approval, like the process in Japan (15). The limited availability of this medication may discourage physicians from prescribing it and could partially explain the lower usage observed in our study.

This study presents the longest epidemiological follow-up of LEMS reported to date, utilizing a comprehensive national database encompassing the entire Turkish population. However, several limitations should be acknowledged. Due to heterogeneity and incomplete documentation within electronic medical records, data on presenting symptoms, individual risk factors (e.g., smoking history or environmental exposures), electrophysiological findings, antibody status, details of treatment protocols, and long-term outcomes could not be evaluated. As a result, detailed clinical phenotyping and treatment response analysis were beyond the scope of this study. Future prospective, multicenter studies incorporating clinical, serological, and neurophysiological data are needed to further elucidate disease mechanisms, progression patterns, and treatment outcomes across different LEMS subtypes.

In conclusion, this is the first nationwide epidemiological study of LEMS in Türkiye utilizing the national electronic health database, and one of the few large-scale, population-based studies reported in the literature. The annual incidence of LEMS ranged from 0.09 to 0.30 per million between 2015 and 2024, while the prevalence of LEMS was 1.11 per million in 2024. Both incidence and prevalence of LEMS in Türkiye were found to be lower than those reported in other countries, including the Netherlands, the United States, Japan, and Ireland. Although the incidence of P-LEMS exceeded that of A-LEMS, the prevalence of A-LEMS was higher, likely reflecting its more favorable survival profile. Consistent with prior research, P-LEMS was more common in older males and predominantly associated with SCLC, whereas A-LEMS occurred more frequently in younger females. This study provides valuable epidemiological data on LEMS from Türkiye, enriching the global understanding of the disease.

## Data Availability

The datasets presented in this article are not readily available because data are available from the corresponding author only with the permission of Republic of Türkiye Ministry of Health due to legal legislations. Requests to access the datasets should be directed to BI, berin.inan@yahoo.com.

## Footnotes

^1^ www.graphpad.com

## References

[1] Punga AR Maddison P Heckmann JM Guptill JT Evoli A. Epidemiology, diagnostics, and biomarkers of autoimmune neuromuscular junction disorders. Lancet Neurol. (2022) 21:176–88. doi: 10.1016/S1474-4422(21)00297-0, PMID: 35065040

[2] Titulaer MJ Maddison P Sont JK Wirtz PW Hilton-Jones D Klooster R. Clinical Dutch-English Lambert-Eaton Myasthenic syndrome (LEMS) tumor association prediction score accurately predicts small-cell lung cancer in the LEMS. J Clin Oncol. (2011) 29:902–8. doi: 10.1200/JCO.2010.32.0440, PMID: 21245427

[3] Odabasi Z Demirci M Kim DS Lee DK Ryan HF Claussen GC. Postexercise facilitation of reflexes is not common in Lambert-Eaton myasthenic syndrome. Neurology. (2002) 59:1085–7. doi: 10.1212/wnl.59.7.1085, PMID: 12370470

[4] Ivanovski T Miralles F. Lambert-Eaton Myasthenic syndrome: early diagnosis is key. Degener Neurol Neuromuscul Dis. (2019) 9:27–37. doi: 10.2147/DNND.S192588, PMID: 31191084 PMC6524763

[5] Weinberg DH. Lambert-Eaton myasthenic syndrome: Clinical features and diagnosis 2023 (2025). Available online at: https://www.uptodate.com/contents/lambert-eaton-myasthenic-syndrome-clinical-features-and-diagnosis?search=lambert%20eaton%20miyastenik%20sendromu&source=search_result&selectedTitle=1%7E33&usage_type=default&display_rank=1 (Accessed June 04, 2025).

[6] Payne M Bradbury P Lang B Vincent A Han C Newsom-Davis J. Prospective study into the incidence of Lambert Eaton myasthenic syndrome in small cell lung cancer. J Thorac Oncol. (2010) 5:34–8. doi: 10.1097/JTO.0b013e3181c3f4f1, PMID: 19934775

[7] Wirtz PW Wintzen AR Verschuuren JJ. Lambert-Eaton myasthenic syndrome has a more progressive course in patients with lung cancer. Muscle Nerve. (2005) 32:226–9. doi: 10.1002/mus.20332, PMID: 15793845

[8] Liu Y Xi J Zhou L Wu H Yue D Zhu W. Clinical characteristics and long term follow-up of Lambert-Eaton myasthenia syndrome in patients with and without small cell lung cancer. J Clin Neurosci. (2019) 65:41–5. doi: 10.1016/j.jocn.2019.04.003, PMID: 31072737

[9] Maddison P Lipka AF Gozzard P Sadalage G Ambrose PA Lang B. Lung cancer prediction in Lambert-Eaton myasthenic syndrome in a prospective cohort. Sci Rep. (2020) 10:10546. doi: 10.1038/s41598-020-67571-9, PMID: 32601396 PMC7324357

[10] Wirtz PW Dijk JG Doorn PA Engelen BG Kooi AJ Kuks JB. The epidemiology of the Lambert-Eaton myasthenic syndrome in the Netherlands. Neurology. (2004) 63:397–8. doi: 10.1212/01.wnl.0000130254.27019.14, PMID: 15277653

[11] Abenroth DC Smith AG Greenlee JE Austin SD Clardy SL. Lambert-Eaton myasthenic syndrome: epidemiology and therapeutic response in the national veterans affairs population. Muscle Nerve. (2017) 56:421–6. doi: 10.1002/mus.25520, PMID: 27997683

[12] TUIK. Nüfus ve Demografi. (2025). Available online at: https://data.tuik.gov.tr/Kategori/GetKategori?p=Nufus-ve-Demografi-109 (Accessed July 5, 2025).

[13] Wirtz PW Nijnuis MG Sotodeh M Willems LN Brahim JJ Putter H. The epidemiology of myasthenia gravis, Lambert-Eaton myasthenic syndrome and their associated tumours in the northern part of the province of South Holland. J Neurol. (2003) 250:698–701. doi: 10.1007/s00415-003-1063-7, PMID: 12796832

[14] Lefter S Hardiman O Ryan AM. A population-based epidemiologic study of adult neuromuscular disease in the Republic of Ireland. Neurology. (2017) 88:304–13. doi: 10.1212/WNL.0000000000003504, PMID: 27927941

[15] Yoshikawa H Adachi Y Nakamura Y Kuriyama N Murai H Nomura Y. Nationwide survey of Lambert-Eaton myasthenic syndrome in Japan. BMJ Neurol Open. (2022) 4:e000291. doi: 10.1136/bmjno-2022-000291, PMID: 36110924 PMC9445827

[16] Lipka AF Boldingh MI van Zwet EW Schreurs MWJ Kuks JBM Tallaksen CM. Long-term follow-up, quality of life, and survival of patients with Lambert-Eaton myasthenic syndrome. Neurology. (2020) 94:e511–20. doi: 10.1212/WNL.0000000000008747, PMID: 31831596 PMC7080283

[17] Govindan R Page N Morgensztern D Read W Tierney R Vlahiotis A. Changing epidemiology of small-cell lung cancer in the United States over the last 30 years: analysis of the surveillance, epidemiologic, and end results database. J Clin Oncol. (2006) 24:4539–44. doi: 10.1200/JCO.2005.04.4859, PMID: 17008692

[18] Howlader N Forjaz G Mooradian MJ Meza R Kong CY Cronin KA. The effect of advances in lung-Cancer treatment on population mortality. N Engl J Med. (2020) 383:640–9. doi: 10.1056/NEJMoa1916623, PMID: 32786189 PMC8577315

[19] Desai MK Brinton RD. Autoimmune disease in women: endocrine transition and risk across the lifespan. Front Endocrinol. (2019) 10:265. doi: 10.3389/fendo.2019.00265, PMID: 31110493 PMC6501433

[20] Wirtz PW Willcox N van der Slik AR Lang B Maddison P Koeleman BP. HLA and smoking in prediction and prognosis of small cell lung cancer in autoimmune Lambert-Eaton myasthenic syndrome. J Neuroimmunol. (2005) 159:230–7. doi: 10.1016/j.jneuroim.2004.10.018, PMID: 15652424

[21] Titulaer MJ Wirtz PW Kuks JB Schelhaas HJ van der Kooi AJ Faber CG. The Lambert-Eaton myasthenic syndrome 1988-2008: a clinical picture in 97 patients. J Neuroimmunol. (2008) 201:153–8. doi: 10.1016/j.jneuroim.2008.05.025, PMID: 18644631

[22] Wirtz PW Bradshaw J Wintzen AR Verschuuren JJ. Associated autoimmune diseases in patients with the Lambert-Eaton myasthenic syndrome and their families. J Neurol. (2004) 251:1255–9. doi: 10.1007/s00415-004-0528-7, PMID: 15503107

[23] Wirtz PW Smallegange TM Wintzen AR Verschuuren JJ. Differences in clinical features between the Lambert-Eaton myasthenic syndrome with and without cancer: an analysis of 227 published cases. Clin Neurol Neurosurg. (2002) 104:359–63. doi: 10.1016/s0303-8467(02)00054-9, PMID: 12140105

[24] Wirtz PW Verschuuren JJ van Dijk JG de Kam ML Schoemaker RC van Hasselt JG. Efficacy of 3,4-diaminopyridine and pyridostigmine in the treatment of Lambert-Eaton myasthenic syndrome: a randomized, double-blind, placebo-controlled, crossover study. Clin Pharmacol Ther. (2009) 86:44–8. doi: 10.1038/clpt.2009.35, PMID: 19357643

[25] Weinberg DH. Lambert-Eaton myasthenic syndrome: Treatment and prognosis 2024, (2024). Available online at: https://www.uptodate.com/contents/lambert-eaton-myasthenic-syndrome-treatment-and-prognosis?search=treatment%20of%20LEMS&source=search_result&selectedTitle=1~33&usage_type=default&display_rank=1#H1628431462 (Accessed June 04, 2025).

[26] Oh SJ. Amifampridines are the Most effective drugs for treating Lambert-Eaton Myasthenic syndrome with a focus on pediatric Lambert-Eaton Myasthenic syndrome. J Clin Neurol. (2024) 20:353–61. doi: 10.3988/jcn.2024.0018, PMID: 38951970 PMC11220352

[27] Oh SJ. Amifampridine for the treatment of Lambert-Eaton myasthenic syndrome. Expert Rev Clin Immunol. (2019) 15:991–1007. doi: 10.1080/1744666X.2020.1670061, PMID: 31533480

[28] Meisel A Sieb JP Le Masson G Postila V Sacconi S. The European Lambert-Eaton Myasthenic syndrome registry: long-term outcomes following symptomatic treatment. Neurol Ther. (2022) 11:1071–83. doi: 10.1007/s40120-022-00354-8, PMID: 35511347 PMC9338181

